# Modulation of microRNA 20b with resveratrol and longevinex is linked with their potent anti-angiogenic action in the ischaemic myocardium and synergestic effects of resveratrol and γ-tocotrienol

**DOI:** 10.1111/j.1582-4934.2011.01480.x

**Published:** 2012-09-26

**Authors:** Partha Mukhopadhyay, Somak Das, Md Kaimul Ahsan, Hajime Otani, Dipak K Das

**Affiliations:** a Laboratory of Physiologic Studies, National Institute on Alcohol Abuse and Alcoholism, National Institutes of HealthBethesda, MD, USA; b Cardiovascular Research Center, University of Connecticut School of MedicineFarmington, CT, USA; c Yale University, Section of Digestive DiseasesNew Haven, CT, USA; d Department of Internal Medicine, Kansai Medical UniversityMoriguchi, Osaka, Japan

**Keywords:** ischaemia/reperfusion, redox, cardiovascular, microRNA, resveratrol/longevinex, γ-tocotrienol

## Abstract

Resveratrol, a constituent of red wine, and γ-tocotrienol, a constituent of palm oil are important for cardioprotection. Although microRNAs are known regulators for genes involved in cardiac remodelling, the regulatory pathway involving microRNA has not been studied so far. We explored the cardioprotection by resveratrol, longevinex and γ−tocotrienol in ischaemia/reperfusion(I/R) model of rat and determined miRNA profile from isolated RNA. Systemic analyses of miRNA array and theirs targets were determined using a number of computational approaches. Resveratrol and γ-tocotrienol, either alone or in combination, modulated the expression pattern of miRNAs close to the control level based on PCA analyses. Differential expression was observed in over 75 miRNAs, some of them, such as miR-21 and miR-20b (anti-angiogenic) were previously implicated in cardiac remodelling. The target genes for the highest differentially expressed miRNA include genes of various molecular functions such as TGFβ1–Smad3 signalling pathway, inflammation and their transcription factors, which may play key role in reducing I/R injury. Administration of antagomiR-20 attenuated I/R induced vascular endothelial growth factor and HIF1α level. All the interventions treated for 3 weeks lead to significant cardioprotection against ischaemia/reperfusion injury. A unique signature of miRNA profile is observed in control heart pretreated with resveratrol or γ-tocotrienol. We have determined specific group of miRNA in heart that have altered during IR injuries. Most of those altered microRNA expressions modulated close to their basal level in resveratrol or longevinex treated I/R rat. Interestingly, resveratrol and γ-tocotrienol resulted in synergestic action.

## Introduction

Human diet consists of a wide number of plant-derived polyphenolic compounds, which may protect against cardiovascular diseases, neurodegeneration and inflammatory response in aging and viral infections. Resveratrol, a microcomponent in red wine, is a naturally occurring phenol belonging to stilbene family of compound. Resveratrol was first found as an anti-proliferative agent for cancer [1]. Overwhelming epidemiological and experimental evidence documents resveratrol as a cardioprotective agent [2, 3]. Recent scientific advancement suggests that resveratrol is beneficial against diverse cardiac diseases including ischaemic heart disease, hypertrophy, heart failure, atherosclerosis, hypertension, diabetes and obesity [4, 5]. Similarly, another plant-derived compound γ-tocotrienol, member of Vitamin E family, has been implicated in exerting beneficial effect in cardiovascular aging, diabetes and anti-angiogenesis [6–8] The most prominent way resveratrol functions is probably through its ability to perform intracellular signalling and alter gene expression. Resveratrol can alter a variety of genes thereby changing the ‘death signal’ into a ‘survival signal’ [9]. The most prominent mechanism appears to be its ability to induce several longevity genes including *Sirt1*, *Sirt3*, *Sirt4*, *FoxO1*, *Foxo3a* and *PBEF* [5]. Thus resveratrol prevents aging-related decline in cardiovascular function without affecting actual survival or life span of mice [5]. Thus, resveratrol and γ-tocotrienol possess a myriad of beneficial effects and can act at multiple levels, such as cellular signalling, enzymatic pathways, apoptosis and gene expression.

The gene regulation of resveratrol through micoRNA at the molecular level in ischaemic heart has recently been demonstrated [10]. MicroRNAs are endogenous small RNAs that play regulatory roles targeting mRNA for mostly translational repression and occasionally translational activation [11].

Cardioprotective abilities of resveratrol and/or longevinex are intimately linked to the regulation of genes, and they display unique miRNA expression pattern. A recent study showed that ischaemia/reperfusion either down-regulates or up-regulates large number of miRNAs, which are restored with resveratrol and/or longevinex [10]. Differential expression was observed in over 75 miRNAs, especially for microRNA 20b (miR-20b), which demonstrated significant modulation. Because the angiogenic gene vascular endothelial growth factor (VEGF) is the target gene for miR-20b, we hypothesized that resveratrol, especially longevinex, would display anti-angiogenic properties. Indeed, a recent study showed anti-angiogenic effect of resveratrol in a swine model of metabolic syndrome [12]. Additionally, a recent paper demonstrated synergistic effects of resveratrol with γ-tocotrienol, which also possesses potent cardioprotective abilities [13]. The present study was designed to examine the effects of resveratrol/longevinex with or without γ-tocotrienol in the ischaemic myocardium on hemodynamic functions and angiogenic factors VEGF and HIF-1α. Our results demonstrated that longevinex indeed possesses potent anti-angiogenic action on the heart, which corroborated with its ability to down-regulate VEGF and HIF-1α. Here, we have also demonstrated that antagomir specific for miRNA 20b reversed the anti-angiogenic action of resveratrol and longevinex.

## Materials and methods

### Animals

All animals used in this study received humane care in compliance with the regulations relating to animals and experiments involving animals and this adheres to the principles stated in the Guide for the Care and Use of Laboratory Animals, NIH Publication, 1996 edition, and all the protocols (Proposal # 2008-484) were approved by the Institutional Animal Care Committee of University of Connecticut Health Center, Farmington, CT, USA. Male Sprague-Dawley rats weighing between 250 and 300 g were fed *ad libitum* regular rat chow with free access to water until the start of the experimental procedure. Animals were gavaged with either resveratrol (5 mg/kg/day) (Sigma-Aldrich, St. Louis, MO, USA) or longevinex (100 mg/kg/day) (Longevinex Inc, North Las Vegas, NV, USA) or γ-tocotrienol (5 mg/kg/day), alone or in combination with resveratrol (5 mg/kg/day) for 21 days. Previous studies from our laboratory established the appropriate dose and time periods for each compound used in this experiment [14, 15]. Commercial formulation in longevinex contains Trans resveratrol from Polygonum cuspidatum, 100 mg (micronized, microencapsulated) Quercetin 25 mg IP6 calcium phytate from rice bran 75 mg Vitamin D3, 1000 IU ferulic acid from rice bran 25 mg. Each capsule contains 303.9 mg of ingredients and considered as 100 mg longevinex (equivalent to 100 mg resveratrol) in this study.

Isolated working heart preparation and determination of cardiac function were performed as described previously [5].

Cardiomyocyte apoptosis and infarct size estimation were assessed as described previously [5, 16]. ‘Detailed Method’ is described in Supporting Information.

### MicroRNA isolation and cDNA preparation

Total RNA from rat heart samples were isolated using Trizol reagent (Invitrogen, Life Technologies, Grand Island, NY, USA) and further purified using mirVANA miRNA isolation kit (Ambion, Life Technologies, Grand Island, NY, USA) [17, 18]. cDNAs were prepared using Taqman miRNA Reverse Transcription kit and Megaplex Rodent Pool A and B primers sets.

### Profiling of miRNA expression

miRNA expression profiling were carried out using quantitative real-time PCR method by TaqMan® Gene Signature Rodent Arrays on a 384-well microfluidic card in 7900HT Real-time PCR machine (Applied Biosystems, Foster City, CA, USA) according to manufacturer’s recommendation. Each miRNA was quantified by two specific amplicon primers and one specific probe. Comprehensive coverage of Sanger miRBase v10 was enabled across a two-card set of TaqMan® MicroRNA Low Density Arrays (TLDA Array A and B) for a total of 518, and 303 unique assays, specific to rat miRNAs, respectively. In addition, each array contains six control assays – five carefully selected candidate endogenous control assays, and one negative control assay. Profiling of miRNA by array has been used previously [10, 19].

### Analyses of miRNA gene expression data

Real-time PCR data expressed as Ct values from array A and B were combined using R script (provided by GeneSpring Informatics Support Team) and processed using GeneSpringGX 11.5 software (Agilent Technologies, Santa Clara, CA, USA). After analysis, 588 entities were detected from array A and B. All statistical analyses including normalization to endogenous control, quality control, filtering, correlation analyses and principal component analyses were carried out by GeneSpring GX software. Individual Taqman assay for mmu-miR-20b were purchased from ABI and performed according to manufacturer recommendation and MamnU6 (ABI) used as endogenous control.

### miRNA target prediction

miRNA targets have been predicted using TargetScan in-built and plugged within GeneSpring GX software 11.5.

### Effects of antagomir miR-20b on the cardioprotection and the expression of HIF-1α and VEGF

Since all our interventions including the treatments with resveratrol, longevinex and γ-tocotrienol indicated several-fold up-regulation of miRNA 20b, we used antagomir mirRNA20b to specifically examine the role of miRNA 20b on the cardioprotective effects of these compounds. The animals were treated with antagomir miRNA20b (i.v.) 72 hrs prior to the experiment. Phosphate buffered saline was used as vehicle (i.v.). After 72 hrs, all animals were sacrificed and myocardial function was determined and Western blot analysis was performed.

### Assessment intracellular ROS with CM-H2DCFDA

Since resveratrol functions by changing ischaemia/reperfusion-mediated harmful oxidative environment into a reduced environment, intracellular ROS concentration was determined with CM-H_2_DCFDA (5-(and-6)-chloromethyl-2′, 7′-dichlo-rodihydrofluorescein diacetate, acetyl ester) (10 μM; Molecular Probes, Eugene, OR, USA), a derivative of DCF-DA, with an additional thiol reactive chloromethyl group, which enhances the ability of the compound to bind to intracellular components, thereby prolonging the dye’s cellular retention. The dye was injected intravenously, prior to induction of ischaemia/reperfusion, and at the end of the experiments, the level of fluorescence was determined for the generation of Reactive Oxygen Species (ROS) by measuring the fluorescent oxidation product CM-DCF in the cytosol, at an excitation wavelength of 480 nm and an emission wavelength of 520 nm.

### Western blot analysis for HIF-1α and VEGF

The effects of resveratrol, longevinex and γ-tocotrienol on the expression of HIF-1α and VEGF were estimated by Western blot analysis using antibodies against VEGF and HIF-1α. Detailed Method is described in Supporting Information.

### Confocal microscopy imaging techniques and image analysis

Heart tissue samples were collected at the end of experiments, fixed in 2% buffered paraformaldehyde (pH 7.4), embedded and frozen in O.C.T. compound, and subjected to cryosectioning for confocal microscopy [20]. Detailed Method is described in Supporting Information.

### Statistical analysis

The values for myocardial function parameters, infarct size and apoptosis were expressed as the mean ± standard error of mean. A one-way analysis of variance was first carried out to test for any differences in mean values between groups. If differences were established, the values of the resveratrol-treated groups were compared with those of the control group by modified *t*-test. The results were considered significant if *P* < 0.05.

## Results

### Molecular signatures of miRNA in ischaemia-reperfused rat heart

MicroRNA profiles were analysed by TLDA array specific for 588 miRNA and five endogenous control for rat. Two sets of procedure were performed such as sham basal level (BS) and ischaemia-reperfused (IR) rat heart. Rats were pretreated with the following compounds 21 days prior to the procedure: (*i*) Vehicle (VEH), (*ii*) resveratrol (RES), (*iii*) γ-tocotrienol (TOC) and (*iv*) resveratrol and γ-tocotrienol (RES+TOC). Thus, arrays were carried out from eight different groups. RNAs were isolated after 30 min. ischaemia and 2-hr reperfusion of the heart from IR samples or from sham vehicle (BS) samples processed the same way without ischaemia and reperfusion. The normalized Ct values for each miRNA were shown in profile plot (Fig. S1A).

**Fig 1:**
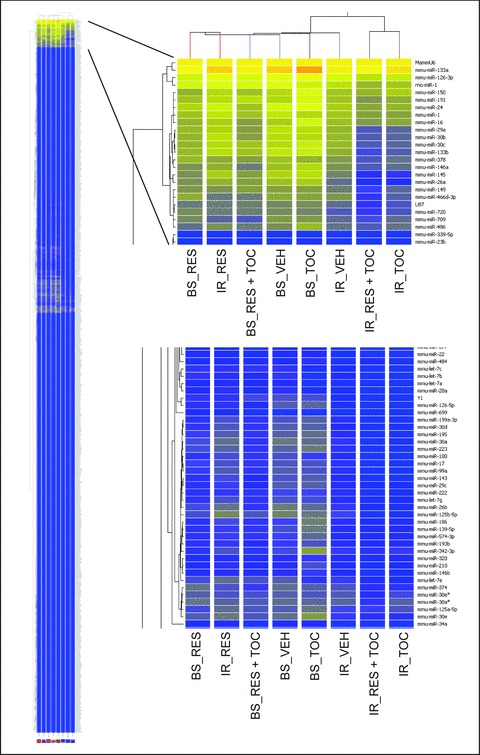
Global miRNA expression and hierarchical clustering of sham vehicle (BS) or ischaemia-reperfused (IR) hearts pretreated with resveratrol (RES), tocotrienol (TOC) and resveratrol and tocotrienol (RES + TOC) using Taqman Microfluidics Array card. A partial heat map depicts the distinct patterns of miRNA expression in the samples. Vertical columns and horizontal rows represent samples and miRNAs, respectively. The red or yellow colour represents a relatively high or low expression, respectively. An overall expression pattern of 588 miRNAs is shown by a compressed heat map (left).

To explore the relation of differentiated state and the pattern of miRNA expression, we constructed a hierarchical tree based on the similarity of miRNA expression profiles on sample and entity (Fig. 1). A group structure emerged in the unsupervised dendogram analysis indicating groups of samples according to patterns of miRNA expression. The heat map in Figure 1 shows the detected miRNAs displaying highest variance. From these miRNAs, two general patterns were observed. The expression of all detected miRNAs with greater than 5-fold expression between Sham vehicle and I/R sample is displayed in Table 1. The miR name with asterisk sign indicates the miRNA is non-dominant form from the miRNA precursor. 5p is from the 5′ end of the precursor; 3p is from the 3′ end of the precursor as described for some miRNA.

**Table 1:** miRNAs expressed as relative fold change compared to basal level in vehicle group

Up-regulated miRNA	IR	Down-regulated miRNA	IR
mmu-miR-704	1848	mmu-miR-361	-84
rno-miR-207	65	mmu-miR-10b	-81
rno-miR-346	56	mmu-miR-297a*	-76
mmu-miR-335–3p	53	mmu-miR-376a	-72
mmu-miR-744*	44	mmu-miR-692	-69
mmu-miR-337–3p	44	mmu-miR-208	-68
rno-miR-493	43	mmu-miR-467c	-65
mmu-miR-433	33	mmu-miR-804	-62
mmu-miR-330*	32	mmu-miR-137	-53
mmu-miR-134	31	mmu-miR-197	-41
mmu-miR-666–5p	31	mmu-miR-741	-36
mmu-miR-21*	29	mmu-miR-296–5p	-36
rno-miR-466b	27	mmu-miR-582–5p	-35
mmu-miR-193*	21	mmu-miR-369–5p	-35
mmu-miR-142–5p	20	mmu-miR-375	-34
mmu-miR-124	11	mmu-miR-337–5p	-34
mmu-miR-327	10	mmu-miR-15a*	-34
mmu-miR-451	9	mmu-miR-331–5p	-34
mmu-miR-411	9	mmu-miR-201	-33
rno-miR-743b	8	mmu-miR-532–5p	-33
mmu-miR-10a*	8	mmu-miR-183	-32
mmu-miR-362–3p	8	mmu-miR-376b*	-27
mmu-miR-429	8	mmu-miR-186*	-17
rno-miR-327	8	mmu-miR-721	-16
mmu-miR-452	8	mmu-miR-351	-16
mmu-miR-682	8	mmu-miR-34c	-16
mmu-miR-335–5p	8	mmu-miR-542–3p	-14
rno-miR-758	8	mmu-miR-188–5p	-11
mmu-miR-598	8	mmu-miR-23b	-11
mmu-miR-196b	7	mmu-miR-126–5p	-10
mmu-miR-672	6	mmu-miR-20b	-10
mmu-miR-27a*	6	mmu-miR-27b	-8
rno-miR-743a	5	mmu-miR-503	-8
		mmu-miR-181c	-8
		mmu-miR-21	-7
		mmu-miR-17*	-7
		rno-miR-532–5p	-7
		mmu-let-7i	-7
		mmu-miR-206	-6
		mmu-miR-301b	-6
		mmu-miR-500	-5
		mmu-miR-28	-5

Using principal component correlation analysis, we compared the overall miRNA expression profiles by studying the closest and furthest samples of all groups (Fig. S1B). As expected, the overall miRNA expression levels of resveratrol, γ-tocotrienol or their combination in I/R samples showed significantly higher correlations with sham samples. Sham samples of resveratrol and γ-tocotrienol have complete distant profile, however they are quite similar in I/R group.

### Pathway analyses of target genes of miRNA in ischaemic heart

**Fig 2:**
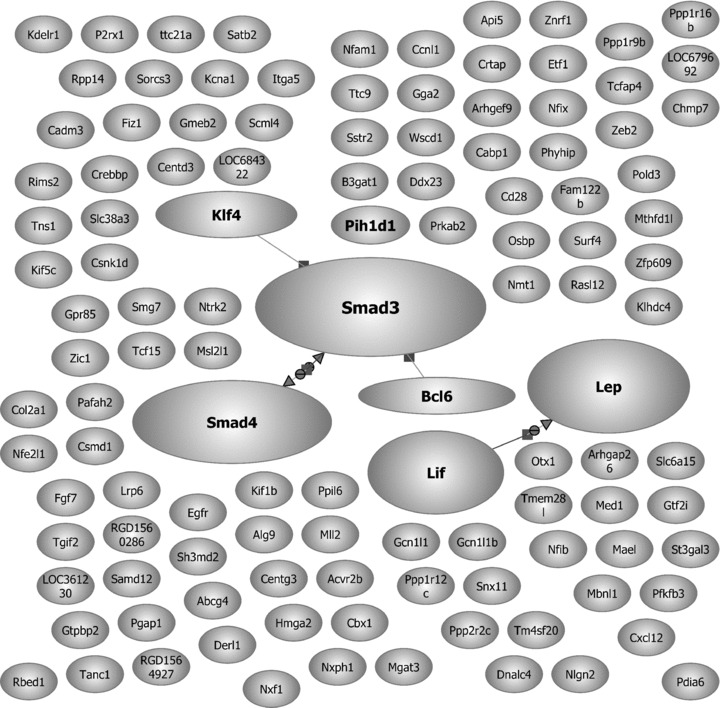
MicroRNA interaction network for target genes. Target genes for microRNAs (50-fold or higher in IR samples) were analysed for pathways only through direct interactions. Target genes of 117 leads to only two significant networks as shown in schematic diagram.

To explore the likely biological consequences of I/R-mediated miRNA expression changes, we identified mRNA transcripts containing predicted target sites for these miRNAs, which were either up or down 50-fold or more in ischaemic heart. Since predicted sites of any one miRNA can be counted in the hundreds it was necessary to reduce these numbers to a manageable and biologically meaningful size, we selected a cut-off of 50-fold. We used the TargetScan algorithm for our predictions due in part to our earlier successes in predicting target gene modulation and we used only evolutionary conserved targets. Applying more stringent context score cut-offs increases the likelihood of observing biologically relevant effects when miRNAs are exogenously added *in vitro*. Pathway analyses were carried out with target genes of miRNA. TargetScan analyses result in 117 genes and two pathways were found to be significant (Fig. 2). The important candidates of the pathways appeared to be Smad family members Smad 3 and 4 in addition to BCl6, KIf4, Lif and Lep.

### Synergistic effect of miRNA by resveratrol and γ-tocotrinol in ischaemia-reperfused rat heart

Based on principal component analyses, the basal level pattern of resveratrol, γ-tocotrinol and their combination are distinctly different. However, all the three samples are closer entity in I/R samples. Further analyses revealed that a significant number of miRNAs were regulated synergistically for resveratrol and γ-tocotrinol (Table 2).

**Table 2:** Synergistic effect of resveratrol (RES) and γ-tocotrienol (TOC): miRNAs expressed as relative fold change compared to basal level in vehicle group

miRNA	RES	TOC	RES+TOC				
mmu-miR-142–3p	-1.6	-29.6	-1551.0	**miRNA**	**RES**	**TOC**	**RES+TOC**
rno-miR-352	-3.9	-10.6	-1200.2	mmu-miR-183	-19.2	-53.6	-87.7
mmu-miR-136	-2.4	-56.1	-759.5	mmu-miR-376b*	-16.1	-45.0	-73.6
mmu-miR-708	-10.1	-446.7	-730.1	mmu-miR-125a-3p	-10.4	-29.1	-47.6
mmu-miR-200b	-155.3	-433.5	-708.7	mmu-miR-186*	-10.3	-28.8	-47.1
mmu-miR-801	-112.9	-17.6	-515.2	mmu-miR-434–3p	-2.2	-16.9	-45.6
mmu-miR-27b	-1.8	-71.4	-462.7	mmu-miR-214*	-2.6	-5.0	-43.5
mmu-let-7c-1*	-73.2	-204.4	-334.1	mmu-miR-34c	-9.4	-26.1	-42.7
mmu-miR-542–3p	-5.9	-195.6	-319.8	mmu-miR-706	-9.3	-5.0	-38.7
mmu-miR-487b	-2.0	-161.5	-263.9	mmu-miR-467b*	-10.1	-2.2	-33.6
rno-miR-17–3p	-1.6	-152.0	-248.5	mmu-miR-323–3p	-3.7	-23.3	-29.8
mmu-miR-10b	-2.4	-136.6	-223.3	mmu-miR-202–3p	-6.5	-5.9	-21.4
mmu-miR-29b	-3.0	-135.1	-220.9	mmu-miR-339–5p	-1.6	-9.6	-19.6
mmu-miR-297a*	-2.4	-128.4	-209.8	mmu-miR-181c	-2.0	-10.5	-14.6
mmu-miR-692	-41.5	-115.8	-189.2	mmu-miR-203	-4.6	-6.4	-13.8
mmu-miR-208	-40.6	-113.5	-185.5	mmu-miR-467a*	-2.6	-3.9	-11.4
mmu-miR-467c	-38.9	-108.6	-177.5	mmu-miR-214	-1.7	-6.1	-10.9
mmu-miR-137	-31.7	-6.8	-144.8	mmu-miR-29c	-1.8	-10.5	-10.7
rno-miR-532–5p	-2.0	-59.1	-126.9	mmu-miR-466d-3p	-2.7	-4.2	-9.9
mmu-miR-466d-5p	-23.2	-64.7	-105.7	mmu-miR-22	-1.6	-4.6	-9.9
mmu-miR-582–5p	-21.3	-59.4	-97.1	mmu-miR-690	-1.9	-2.1	-9.7
rno-miR-421	-21.3	-59.3	-97.0	mmu-miR-193	-4.9	-3.5	-8.1
mmu-miR-369–5p	-20.9	-58.3	-95.3	mmu-miR-27b*	-2.1	-2.9	-8.0
mmu-miR-684	-20.8	-58.1	-94.9	mmu-miR-378	-1.6	-4.6	-7.7
mmu-miR-375	-20.6	-57.6	-94.2	mmu-miR-9*	-1.9	-18.4	-7.7
mmu-miR-337–5p	-20.5	-57.4	-93.8	mmu-miR-204	-2.5	-5.3	-7.5
mmu-miR-15a*	-20.3	-56.8	-92.8	mmu-miR-28*	-1.9	-3.2	-6.5
mmu-miR-532–5p	-19.8	-55.3	-90.4	mmu-miR-34c*	-3.7	-1.7	-6.5
**mmu-miR-709**	-2.9	-2.4	-4.3	mmu-miR-326	-3.9	-2.9	-5.6
**rno-miR-664**	-1.8	-2.2	-3.6	mmu-miR-350	-1.4	-110.5	-5.1
				mmu-let-7e	1.4	-7.6	-5.1
				mmu-miR-542–5p	-1.9	-5.2	-5.1
				rno-miR-20b-5p	-3.0	-4.5	-5.0
				mmu-miR-374	-1.7	-3.7	-4.4

### Effects of resveratrol, longevinex and γ-tocotrienol on myocardial function, infarct size and cardiomyocyte apoptosis

To understand the cardiovascular benefit of resveratrol in ischaemia/repurfusion, we included longevinex, a commercial formulation of resveratrol by gavage to rat. The results of different treatments, *i.e.* feeding resveratrol, longevinex and tocotrienol, either alone or in combinations, are shown in Table 3. All the treatments improved cardiac output function including aortic flow, coronary flow, LV developed pressure and its first derivative LV_max_dp/dt in the hearts subjected to 30 min. ischaemia followed by 1 or 2 hrs of reperfusion. These compounds also lowered the infarct size and death due to cardiomyocyte apoptosis, as expected. Consistent with the previous findings, resveratrol in combination with γ-tocotrienol exhibited further improvement of cardiac function and lowered infarct size indicating synergetic action. The cardioprotective effects of these compounds were abolished in the hearts treated with antagomir miRNA20b.

**Table 3:** Effects of antagomir 20b on the cardioprotection afforded by γ-tocotrienol, resveratrol, γ-tocotrienol + resveratrol, and longevinex

	Baseline				60 min. reperfusion				120 min. reperfusion				
	CF	AF	LVDP	LVdp/dt	CF	AF	LVDP	LVdp/dt	CF	AF	LVDP	LVdp/dt	IF
Vehicle	24±0.5	50±1	110±4	3130±60	22±2	25±3	80±5	2023±65	18±2	8±1	52±3	1175±56	35±3
Vehicle +A20b	25±0.7	48±0.2	114±7	3040±43	20±0.9	23±2	77±4	1965±44	19±1	7±2	50±4	1065±44	38±2
Tocotrienol (TOC)	25±0.5	51±1	116±2	3227±81	20±1	27±0.8*	95±3*	2312±25	20±2	19±2*	83±4*	1674±56*	27±2*
Tocotrienol +A20b	23±1	49±2	114±4	3049±32	19±2	26±0.5*	93±2*	2433±28*	18±1	19±3*	84±2*	1597±28*	29±1*
Resveratrol (RES)	25±0	7 5±0.9	115±2	3292±66	19±1	32±2*	101±4*	2405±35*	21±1	20±1*	85±3*	1712±67*	25±2*
Resveratrol +A-20b	22±1.1	53±5	111±3	3188±54	18±0.7	33±1*	98±2*	2334±24*	19±0.6	19±2*	84±1*	1678±32*	27±1
TOC +RES	26±0.4	55±1	119±1	3471±65	23±2	37±1*^†^	110±2*^†^	2723±28°^†^	19±2	19±1*	79±4*	1649±34*	19±1*^†^
TOC +RES +A-20b	22±1	53±2	107±5	2956±112	22±3	36±1*^†^	109±1*^†^	2654±67*	18±3	20±2*	81±3*	1723±44*	20±2*^†^
Longevinex	26±0.8	53±0.7	118±5	3310±82	20±3	32±1*	98±2*	2300±41*	20±2	19±1*	88±4*	1823±55*	23±1*
Longevinex +A-20b	22±1.1	50±3	105±4	2876±97	19±1	30+±0.8*	94±4*	2254±34*	20±1	18±1*	80±3*	1711±41*	25±2+

* *P* < 0.02 compared to control, ^†^*P* < 0.05 compared to –A-20b. CF: coronary flow (ml/min.); AF: aortic flow (ml/min.); LVDP: LV developed pressure (mmHg); LVdp/dt: first derivative of developed pressure (mmHg/sec); IF = infarct size.

### Modulation of miR-20b in ischaemic heart and reversed with resveratrol and γ-tocotrienol

**Fig 3:**
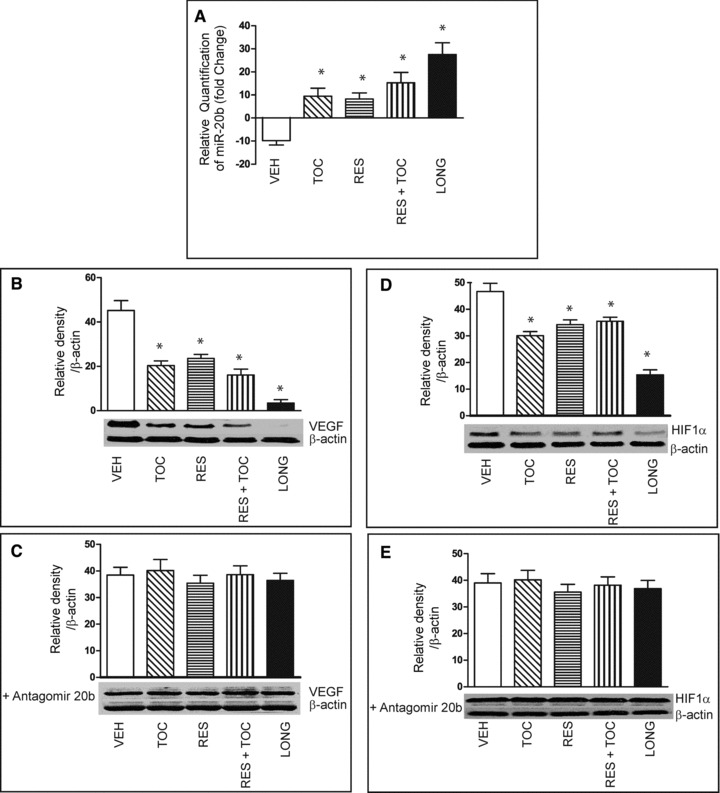
Regulation of miR-20b and the effect of antagomiR-20b on its target VEGF and HIF-1a expression: (A) Taqman real-time PCR quantification of miR-20b in ischaemia experimental groups are IR vehicle (VEH) (1), IR + γ-tocotrienol (TOC) (2), IR + resveratrol (RES) (3), IR + γ-tocotrienol + resveratrol (RES+TOC) (4), and IR + longevinex (LONG) (5). *P < 0.05 *versus* IR vehicle where n = 4/group. All data are normalized and converted to fold change of sham vehicle (BS). (B) VEGF Western blot analyses and its quantification of the above samples. (C) VEGF Western blot analyses and its quantification of the same group of samples when pretreated with antagomiR-20b. *P < 0.05 *versus* IR Vehicle where n = 4/group. (D) VEGF Western blot analyses and its quatification of the samples IR vehicle (VEH) (1), IR + γ-tocotrienol (TOC) (2), IR + resveratrol (RES) (3), IR + γ-tocotrienol + resveratrol (RES+TOC) (4), and IR + longevinex (LONG) (5). (E) VEGF Western blot analyses and its quatification of the same group when pretreated with antagomiR-20b. *P < 0.05 *versus* IR vehicle where n = 4/group.

miR-20b was important for regulation of VEGF in previous studies and also shown to be modulated drastically in ischaemia ischaemia-reperfused rat heart. miR-20b significantly down-regulated in I/R heart as quantified with Taqman real-time PCR (Fig. 3A). The data were presented as fold change compared to sham vehicle control. A down-regulation (9.8-fold) of mir-20b is reversed to 9.4-, 8.2-, 15.2- and 27.5-fold in γ-tocotrinol, resveratrol, resveratrol+gγ-tocotrinol and longevinex pretreated I/R hearts respectively. miR-20b targets HIF1α and also modulates VEGFα expression as reported earlier.

### Effects of antagomir-20b on resveratrol, longevinex and γ-tocotrienol induced expression of HIF-1α and VEGF

The results for the expression of HIF-1α and VEGF by immunoblot were shown in Figure 3B–E. The results indicated that both HIF-1α and VEGF expressions are significantly down-regulated after the treatment. For VEGF, when γ-tocotrienol was used in conjunction with resveratrol, there was further reduction of VEGF expression, suggesting synergistic action. Longevinex resulted in very significant reduction of VEGF expression, far greater than resveratrol and γ-tocotrienol. HIF-1α expression was also reduced with the treatments; however, there were no intergroup differences for reservation and γ-tocotrienol. Again, longevinex displayed greater reduction (compared to resveratrol and γ-tocotrienol) of HIF-1α. Antagomir miRNA20b restored the expressions of both VEGF and HIF-1α for all the treatments suggesting that expressions of VEGF and HIF-1α are dependent of miRNA 20b.

### Assessment of HIF-1α and VEGF with confocal microscopy

**Fig 4:**
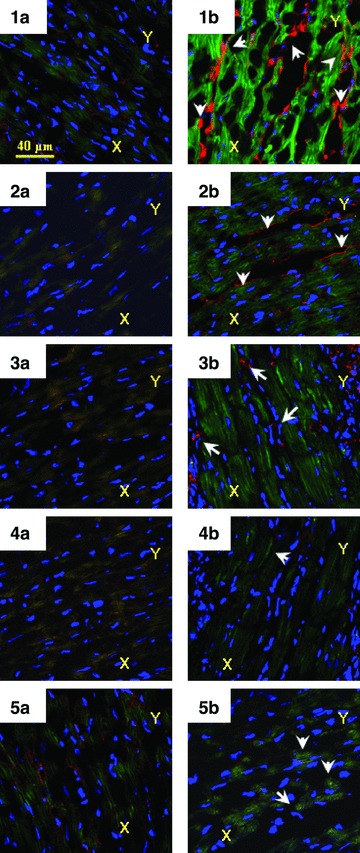
Immunofluorescence imaging of HIF1α and VEGF proteins in sections of rat hearts subjected to ischaemia/reperfusion (I/R) and treatments with γ-tocotrienol, resveratrol or longevinex. Experimentalconditions: Panels (1a-5a) are I/R+vehicle only; Panel 1b is sham treatment; Panel 2b is I/R+γ-tocotrienol; Panel 3b is I/R+ resveratrol; Panel 4b is I/R; γ-tocotrienol+ resveratrol; Panel 5b is I/R+ longevinex. Note: Images of projections of nuclear factor HIF1α and VEGF are shown in green and red respectively; counterstaining of nuclei is presented in blue. Spatial co-localization of HIF1α and VEGF is indicated with white arrows. The confocal images were taken with optical Z-step of 0.5 μm.

Confocal microscopy imaging demonstrated significant increase in HIF1a and VEGF expression in I/R rat heart section (Fig. 4, panels 1a–5a) as compared to sham-treatment (Fig. 4, panel 1b). Note co-localization of immunoreactivity of VEGF and HIF1a is shown in Fig. 4, panels 1a–5a. No such increase has been observed in any of the different treatments, *i.e.*, administration of resveratrol, longevinex and g-tocotrienol, either alone or in combinations (Fig. 4, panels 2b–5b). VEGF is an endothelial cell-specific mitogen *in vitro* and an angiogenic inducer for *in vivo* models. It is now established that VEGF can be up-regulated by HIF1a, a key mediator of hypoxic response. In order to assess the effect of g-tocotrienol and resveratrol on HIF1a and VEGF in ischaemic cardiac tissue, we analyzed immunoreactivity of the expressed polypeptides with profiling of the their relative immunofluorescence in the acquired images shown in Fig. 4. We have demonstrated recently that this technique provides a great deal of unique and reliable information for in-depth-analysis of molecular mechanisms of cell physiology [6].

**Fig 5:**
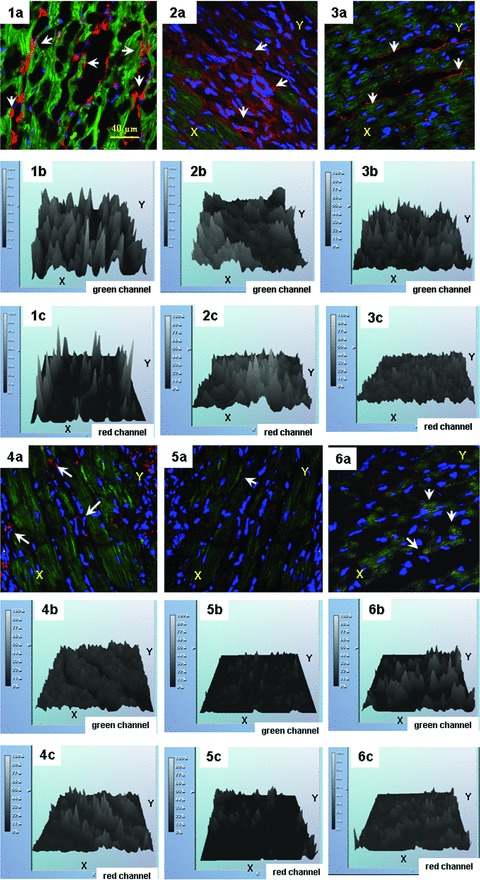
Analysis of Immunofluorescence profiles of HIF1α and VEGF proteins in sections of rat hearts subjected to ischaemia/reperfusion (I/R) and treatments with γ-tocotrienol, resveratrol or longevinex. Experimentalconditions: Panels (1a-1c) are I/R+vehicle only; Panels (2a-2c) are sham treatment; Panels (3a-3c) are I/R + γ-tocotrienol; Panels (4a-4c) are I/R+ resveratrol; Panels (5a-5c) are I/R+ γ-tocotrienol + resveratrol; Panels (6a-6c) are I/R+ longevinex. In the presented panels multichromatic images of projections of nuclear factor HIF1 (green channel), and VEGF (red channel) are shown in Panels a, while the relative immunofluorescence intensities of HIF1α and VEGF are shown in Panels b, and Panels c respectively. Note: counterstainingof nuclei is presented in blue color. Spatial co-localization of HIF1 and VEGF is indicated with white arrows.

The data presented in Figure 4, 1a–5a and quantification in Figure 5, 1a–1c, indicate the presence of relatively high immunoreactivity of HIF1α and VEGF in I/R cardiac tissue where VEGF immunoreactivity was predominantly localized at sites with high microvascular density (co-localization of immunoreactivity of VEGF and HIF-1α is shown in panels 1a–5a). I/R substantially increased levels of immunoreactivity of HIF-1α and VEGF in the injured tissue (Fig. 4, b and Fig. 5, 1a–c). The images shown in Figure 4 panels 2b–4b indicate that both HIF-1α and VEGF expressions are significantly down-regulated after treatment with resveratrol and γ-tocotrienol and a combination thereof. Assessment of relative immunofluorescence of HIF-1α and VEGF (shown in Fig. 5, 2a–c and 3a–c) indicates that there were no intergroup (*e.g.* resveratrol and γ-tocotrienol) differences in the developed effects. However, when γ-tocotrienol was used in conjunction with resveratrol, there was further reduction of HIF-1α and VEGF immunoreactivity, suggesting synergistic action (Fig. 5, 4a–4c). Longevinex resulted in very significant depression of VEGF expression, far greater than resveratrol and tocotrienol (Fig. 4, 5b and Fig. 5, 5a–5c).

### Effects of resveratrol, longevinex and γ-tocotrienol on intracellular ROS activity

**Fig 6:**
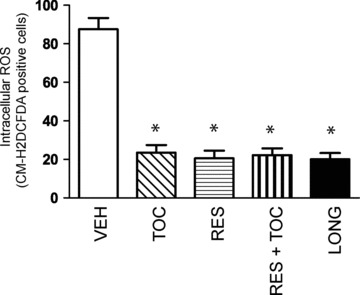
Effects of antagomir 20b on intracellular ROS. Intracellular quantification of ROS by DCFDA in the samples IR vehicle (VEH) (1), IR + γ-tocotrienol (TOC) (2), IR + resveratrol (RES) (3), IR + γ-tocotrienol + resveratrol (RES+TOC) (4), and IR + longevinex (LONG) (5) experimental groups. *P < 0.05 *versus* IR vehicle where n = 4/group.

Intracellular ROS activity determined by monitoring the level of fluorescence by measuring the fluorescent oxidation product CM-DCF in the cytosol is shown in Figure 6. All the compounds including γ-tocotrienol, resveratrol and longevinex lowered intracellular ROS concentration compared to I/R vehicle. However, there was no difference among the treatments.

## Discussion

The results of the present study document that all the treatment groups have superior cardiac performance after 30 min. of ischaemia and 2 hrs of reperfusion. Resveratrol, γ-tocotrienol, and longevinex restored aortic flow, and LV performance as well as infarct size and cardiomyocyte apoptosis towards the control baseline values compared to I/R groups. Consistent with the previous findings, the combined effect of γ-tocotrienol and resveratrol was synergestic. The treatment of the respective groups with antagomir 20b did not change the ventricular performance significantly. Western blot results showed that VEGF and HIF-1α were down-regulated with any of the above treatment and antagomir 20b prevented the down-regulation of the values. Confocal microscopy results support the results of Western blots. Intracellular oxygen radicals were lowered in all the experimental groups indicating the ability of resveratrol and tocotrienol to change the intracellular oxidative environment into a reduced environment. Most importantly, we have identified a pattern of miRNA unique to ischaemia reperfused heart and also in pre-treated hearts with resveratrol, γ-tocotrienol or their combination. MicroRNA has significant role in cardiac remodelling after ischaemic reperfusion. Some microRNAs described in the Table 1 have already implicated in various physiological processes. We have searched specifically the functional evidence in the literature for miRNAs, which are modulated at significant level in I/R. Among up-regulated miRNAs, miR-330 regulates CD44 and CDC42 and thus modulates tumourigenesis and angiogenesis in cancer [21]. miR-134 regulates SIRT1 *via* post-transcriptional regulation of cAMP response binding protein (CREB) expression in brain [22]. miR-142-5p, 5’ end of the precursor of miR-142, is increased in chronic heart failure patients [23]. miR-124, known as tissue injury plasma biomarker, is also increased in I/R hearts [24]. miR-327 is increased in myocardial microvascular endothelial cells in impaired angiogenesis of type 2 diabetic Goto-Kakizaki rats [25]. Among the down-regulated miRNAs, miR-10b contributes to retinoic acid-induced differentiation of neuroblastoma cells and fatty acid metabolism in liver [26, 27]. Retinoic acid and its receptor has multiple role in fatty acid metabolism including regulation of cannabinoid receptor 1 [28]. Cannabinoid receptor 1(CB1) also regulates various physiological process including cell proliferation in liver [29]. It is important to note that CB1 activation and downstream signalling is key regulator in various heart failure models [30, 31]. Among other miRNA such as miR-208 is associated with adverse clinical outcomes in human dilated cardiomyopathy [32]. In type 2 diabetes, plasma levels of miR-15a decreased in patients [33]. There were several miRNAs involved in regulation of G-protein coupled receptors and differentially expressed in MMP-9KO cardiomyocytes including down-regulated miR-376b as we have observed in I/R heart [34] and MMP-9 gene ablation leads to cardiomyocyte dysfunction.

Evolutionary conserved cellular targets of miRNA and their interacting partners are the key regulators of cardiac remodelling. Based on targets of 50-fold or higher changes in miRNA of ischaemia samples, two pathways appeared including Smad3/4. TGFβ1–Smad3 signalling pathway is well known for its role in fibrosis [35]. Lif, a member of the other pathway, was shown to be the most prominently identified in neurons in cerebral ischaemia [36]. The other targets of miRNA, which were described in Figure 2, have potential roles in cardiac modelling including inflammation, fibrosis, neurotrophin and their transcriptional regulation.

Combinations of resveratrol and γ-tocotrienol showed additive or synergistic effects. Their joint actions allowed for many miRNAs showing some synergistic effects. All synergistically effected miRNAs are down-regulated and down-regulated miRNAs will be beneficial due to the fact that down-regulated miRNAs will lead to the up-regulation of their target proteins which may play crucial role in cardiac remodelling [11]. However, detailed study determining their exact role is crucial and will be investigated in future.

Dietary phytochemicals are rapidly becoming popular as functional foods and play a major role for the nutritional supplements. The most significant of these phytochemicals are probably resveratrol (3,4′, 5-trihydroxy-transstillbene), a polyphenolic phytoalexin, present in grapes, wines and peanuts, and tocotrienols (a group of vitamin E isomers) present in large amounts in palm oil and rice bran oil [35, 37]. Both resveratrol and γ-tocotrienol possess potent cardioprotective properties. There is a striking similarities of mechanisms of actions between resveratrol and γ-tocotrienol; both function by changing the intracellular oxidative environment into a reducing environment and by inducing an anti-apoptotic survival signal through PI-3-kinase and Akt and both provide cardioprotection only at lower doses [38, 39]. Consistent with the previous findings that resveratrol and tocotrienol can act synergistically providing greater degree of cardioprotection [13] and co-ordinated autophagy with resveratrol and γ-tocotrienol confers synergetic cardioprotection [13], the present results also demonstrated synergism in LV recovery and lowering of infarct size and cardiomyocyte apoptosis. Resveratrol and γ-tocotrienol can act synergistically providing greater degree of cardioprotection through the induction of Akt-Bcl-2 survival pathway and enhancement of autophagy [13]. At relatively low concentrations, resveratrol (2.5–5.0 mg/kg) and γ-tocotrienol (0.3 mg/kg) induce autophagy as evidenced by their abilities to induce autophagic marker proteins LC3-II and Beclin-I. For γ-tocotrienol, induction of autophagy was more dependent on mTOR while resveratrol-induced autophagy was more independent on mTOR pathway. When resveratrol and γ-tocotrienol were simultaneously used, significant and more pronounced induction of LC3-II formation occurred compared to either resveratrol or γ-tocotrienol alone indicating synergistic effects of these compounds for autophagy. The intention of the present study was not to determine the reasons for the synergistic effects of these two compounds, but to determine the role of microRNAs in the synergism.

Interestingly enough, similar to the reports of the previous studies, the modified resveratrol, longevinex, showed more potent cardioprotective action and more potent anti-angiogenic effects on heart as evidenced by the down-regulation of VEGF and HIF-1α. Since previous studies demonstrated hormetic action of resveratrol [38], cardioprotective at lower doses and harmful at higher doses, generating an inverted U-shape curve or a J-shape curve, we compared the results of resveratrol with a resveratrol formulation, longevinex, which did not exhibit hormesis [35, 40]. Not only longevinex exhibited down-regulation of VEGF and HIF-1α, it also showed many-fold induction of microRNA 20b, a potent anti-angiogenic factor compared to that for resveratrol. Longevinex contains quercetin and ferulic acid in addition to main ingredient resveratrol. Both quercetin and ferulic acid are plant-derived flavinoids and phenolic antioxidant molecules, which may function as additive to resveratrol [21, 24]. Quercetin might exert modulatory effects in cardiomyocytes and endothelial cells independent of classical antioxidant capacity through selective actions at different components of a number of protein kinase and lipid kinase signalling cascades such as phosphoinositide 3-kinase, Akt/PKB, tyrosine kinases, protein kinase C and MAP kinases [21]. Cardio-protective effects longevinex compared to other compounds used in the study may be attributed to these additional ingredients, which work as antioxidants, cellular response contributed by signalling or combination of both.

It is interesting to note that even though both resveratrol and tocotrienol inhibited angiogenesis, this is not reflected in cardioprotection. Similar results were found recently where anti-angiogenic function was noted with resveratrol, but functional recovery was not challenged [12]. This is probably due to the effects of resveratrol and γ-tocotrienol to induce nitric oxide [3] and/or adenosine [41]. Both tocotrienol and resveratrol function by simulating preconditioning like effects on heart [3, 41]. Thus, even though resveratrol and γ-tocotrienol possess anti-angiogenic properties, they can protect the heart through preconditioning-like effects. The results of the recent study showed exactly the same effects, and the authors concluded that resveratrol-induced anti-angiogenetic effects were compromised with its ability to increase nitric oxide and other vasodilatory compounds [12]. In the present study, anti-angiogenic properties of resveratrol, especially longevinex, are supported by their abilities to increase microRNAs that target angiogenesis. This also suggests that resveratrol and longevinex possess chemopreventive properties. Indeed, many reports are available in the literature about chemoprevention with resveratrol. Our results appear to show that longevinex is likely to be a potent drug to prevent cancer in addition to cardioprotection.

## Supporting information

**Fig. S1** Global miRNA analyses. (**A**) Profile plot of miRNA array filtered on expression (20.0–100.0)th percentile in the raw data which were normalized based on endogeneous genes. (**B**) Principal component analyses of all samples. This multivariate analysis demonstrated the proximity of pre-treated IR samples to the control (vehicle) samples. BS: baseline; I/R: ischaemia for 30 min. and 2-hr reperfusion; resveratrol (RES), tocotrienol (TOC) and resveratrol and tocotrienol (RES + TOC).
